# Sleep Apnea Syndrome Represents a Risk for Glaucoma in a Veterans' Affairs Population

**DOI:** 10.5402/2011/920767

**Published:** 2011-11-17

**Authors:** Megan Boyle-Walker, Leo P. Semes, Olivio J. Clay, Lei Liu, Patti Fuhr

**Affiliations:** ^1^Optometry Department, Veterans' Affairs Medical Center, Birmingham, AL 35233, USA; ^2^Department of Optometry, School of Optometry, The University of Alabama at Birmingham, 1716 University Boulevard, Birmingham, AL 35294-0010, USA; ^3^Department of Psychology, The University of Alabama at Birmingham, Birmingham, AL 35294-1170, USA

## Abstract

*Purpose*. To determine whether the diagnosis of sleep apnea syndrome (SAS) represents a risk-factor for glaucoma. *Design*. Retrospective records review. *Methods*. Records in an electronic database which exists at the Birmingham, Alabama Veterans' Affairs Medical Center (BVAMC) permit data retrieval and sorting based on diagnostic and procedural codes. Deidentified data of those having had an eye examination and a diagnostic code (ICD-9) for either sleep apnea or glaucoma were included. *Statistical Analyses*. SPSS version 19 was used to produce crosstabs and to conduct a bivariate logistic regression that examined the relationship between SAS and glaucoma. *Results*. A total of 70,960 unique records were included for analysis. Of the 2,725 patients with a diagnosis of sleep apnea, 228 (8.37%) also had a diagnosis of glaucoma. Diagnosis of glaucoma was present in 3,410 patients among 68,235 patients (5.00%) without sleep apnea. Bivariate logistic regression analysis yielded an odds ratio of 1.736 (*P* < 0.001) suggesting that individuals with SAS are more likely to have a coexisting diagnosis of glaucoma than individuals without SAS. *Conclusions*. Results of this investigation suggest that SAS may represent a significant risk factor for glaucoma and this should be considered when managing patients who report that diagnosis.

## 1. Introduction 

Sleep apnea syndrome (SAS), obstructive sleep apnea (OSA) syndrome, or obstructive sleep apnea/hypopnea syndrome (OSAHS) is characterized by repetitive upper airway obstructions during sleep. This may result in snoring or daytime fatigue, for example. Patients, or their sleeping partners, may not bring these signs and symptoms to the attention of ophthalmic practitioners, and, for that reason, screening for, or recognition of, glaucoma may be delayed or escape detection entirely. A range of ophthalmic complications related to sleep-disordered breathing has been summarized recently. The authors define the spectrum of OSA as repeated episodes of upper airway occlusion during sleep combined with symptoms, most commonly excessive daytime fatigue [1]. 

The earliest association of sleep apnea syndrome (SAS) and glaucoma was suggested by Walsh and Montplaisir in 1982 [2]. Their investigation was initiated by the observation of a patient with a family history of glaucoma who also had family members with sleep disturbances. Since that report, associations between SAS and glaucoma have been proposed [3–9] and challenged [10–12]. Inconsistencies among previous studies include such factors as small sample size and lack of similarities in diagnostic criteria for both SAS and glaucoma. 

Upper airway obstructions during sleep result from narrowing of respiratory passages and can last up to two minutes [13]. This may lead to hypoxia and hypercapnia. Sleep apnea is recognized also as a risk factor for cardiovascular and neurovascular disease [13–16]. Sleep apnea and associated disruptions of breathing during sleep might therefore also represent a risk factor for glaucomatous optic neuropathy. 

Glaucoma is characterized by loss of ganglion cells that becomes manifest as nerve fiber layer and optic nerve atrophy. While glaucoma is a multifactorial disease process, SAS has been suggested as a possible risk factor. One hypothesis is that optic nerve blood flow or autoregulation is affected by repetitive prolonged apneas [17]. An alternative hypothesis suggests that optic nerve damage is secondary to SAS-induced arterial hypertension and arteriolar sclerosis or the induced imbalance between nitric oxide (a vasodilator) and endothelin (a vasoconstrictor) [18]. 

Several studies suggest an association between not only the mechanisms of SAS and glaucoma but also coexistence of the conditions in the same patient. The present study was undertaken to determine if in a population of veterans any statistical association could be established. The study population was chosen as it represents a searchable database consisting of a large number of unique records. In addition, the diagnostic codes for both entities, glaucoma and SAS, were entered by those familiar with diagnostic criteria. 

## 2. Methods

### 2.1. Study Design

This study was a retrospective review of diagnostic and procedure codes. These data exist in an electronic database that adds nearly 40,000 unique patient encounters annually. The study design and methods were approved by the BVAMC Institutional Review Board, and all patient information was deidentified before entered for sorting and analysis. 

A computerized search of the BVAMC patient database was requested for all unique patients with a diagnostic code for sleep apnea (ICD-9 codes 327.20, 327. 21, 327.23, 327. 27, 327.29, 780.51, 780.52, 780.57) or glaucoma (any 365.XX code), all unique patients with a diagnosis code for glaucoma (any 365.XX code), and all patients with a code for eye examination (92014, 92004, 92002, 92012) from January 1, 2003, to December 31, 2005. Three years of data were requested because some patients do not attend the VAMC every year; this would then include those patients who may have been on a 2-year follow-up cycle.

Identification of SAS and glaucoma was based solely on diagnostic code which was entered by an appropriate specialty practitioner. However, no attempt to verify the diagnosis of glaucoma on the basis of optic disc appearance or visual field data entered into the process. Similarly, the redundancy of verifying a diagnosis of SAS was not attempted. Patient age was not considered. The mean age of outpatient veterans was 60 years in 2003 (Utilization of Veterans Affairs Medical Care Services by United States Veterans; http://www.hawaii.edu/hivandaids/Utilization%20of%20Veterans%20Affairs%20Medical%20Care%20Services%20by%20US%20Veterans.pdf, accessed November 2, 2011).

### 2.2. Statistical Analysis

Data for this investigation were incorporated into a specially written program in Microsoft Excel that merged and sorted the data. This program replaced the names and social security numbers with a unique identifying number. Crosstabs and logistic regression were performed using SPSS version 19 to yield percentages and an odds ratio.

## 3. Results

From January 1, 2003, through December 31, 2005, 70,960 individuals seen at the BVAMC had their records included. Crosstabs revealed that 3.8% (2,725/70,960) of these individuals had been diagnosed with sleep apnea, and 5.13% (3,638/70,960) had a glaucoma diagnosis. Of the 2,725 individuals with sleep apnea 8.37% (228) had glaucoma compared to only 5.00% (3,410) of the 68,235 individuals without sleep apnea sleep apnea (see Table 1). With such a large sample size, the Chi-square independence test would be significant for small trivial differences. Therefore, a bivariate logistic regression was performed to yield an odds ratio. The odds ratio is a statistic that is unaffected by sample size. These data yielded an odds ratio of 1.736 (95% CI = 1.509–1.996), *P* < .0001. Therefore, the odds of an individual diagnosed with sleep apnea having glaucoma was 1.736 times the odds of individuals without sleep apnea having glaucoma in this patient population. 

Of the 2,497 individuals with sleep apnea without a glaucoma diagnosis, 45% (1,141) had an eye exam through the VA system. These patients may have had eye exams elsewhere and consequently may have a glaucoma diagnosis that was not coded in the VA medical record. Therefore, these data may represent an underestimate of the prevalence of glaucoma. Also, of the 3,638 patients having a diagnosis for glaucoma, 16% (601/3638) were diagnosed without having an eye exam through the VA system. Those diagnoses were entered by a primary care physician at the VA based on such evidence as prescription for an IOP-lowering medication or report from physician outside the VA system. In either event, it is unlikely that the diagnosis of glaucoma would be in doubt.

## 4. Discussion

The population reported here is representative of that of the BVAMC in general. That is, the population was mostly male and over the age of 50 years. Each of these represents population markers for risk of developing glaucoma or sleep apnea. 

Previous studies of the associations between SAS and glaucoma have been inconsistent. Clinical studies have included patients whose primary diagnosis is SAS or among populations with glaucoma [4–7]. Prevalence of both open-angle and normal-tension glaucoma has been reported to be greater among SAS patients than that of the general population in three studies [3, 5, 7]. Similarly, patients whose diagnosis was either open-angle or normal-tension glaucoma were found to have high prevalence of sleep-disordered breathing [6, 8]. We made no attempt to distinguish among the various possible diagnosis of glaucoma. In another recent report of patients from a sleep-apnea population whose mean age was 62, prevalence of glaucoma among 100 consecutive patients was 27% [19]. Results of the present study suggest that while the prevalence of glaucoma is greater among our study population (5.00%) than in the general population which was estimated to be less than 2% [20], it is still higher in the SAS population (8.4%) and consistent with that of Mojon and colleagues (7.2%) much smaller series [5]. Their study identified glaucoma from physical findings (ONH appearance or visual field) or past history and record review.

Specifically, these various studies have built on the initial report of Walsh and Montplaisir who presented data linking familial glaucoma with sleep apnea syndrome in five patients in two different generations [2]. Four of the five were diagnosed with and treated for glaucoma. No data to verify the diagnosis of glaucoma such as optic disc or visual field information was reported. Details of breathing impairment were presented and conclusive to meet the criteria for sleep apnea syndrome (SAS). The authors speculate on the connection between the two disorders as being genetically related but more likely due to IOP fluctuations or secondary hypoxia [2, 15, 17]. 

Indirect evidence supporting sleep apnea as a causative factor in glaucomatous optic neuropathy comes from two reports which found stabilization of visual field progression following the introduction of nasal continuous positive airway pressure (nCPAP) therapy [21, 22]. Standard therapy for SAS is nCPAP. 

Other supporting evidence for a relationship between SAS and the development of glaucoma comes from a study that measured retinal nerve fiber layer (RNFL) thickness with scanning laser polarimetry (GDx) [9]. None of the patients, who were all admitted for the specific purpose of undergoing polysomnography because of suspected SAS, was treated for glaucoma or ocular hypertension. A group of 34 patients and 20 controls who underwent scanning laser polarimetry showed decreased RNFL parameters in patients compared to controls (*P* < 0.05). One possible confounder in that study is that the instrument used did not yet have corneal compensation. Yet, the authors suggest that such factors as hypoxia, hypercapnia and changes in intrathoracic pressure may lead to loss of ganglion cells that would be indicative of glaucomatous damage that was subclinical in their SAS population [9]. More recently, a group of patients with a diagnosis of SAS was examined specifically for RNFL thickness. Using time-domain OCT, the authors reported that these patients, who had not developed visual field defects, had thinner RNFL than controls [22]. Interestingly, the same authors noted reduced oxygen saturation among those with the more severe grades of SAS and thinner RNFL [23]. 

Two other studies have suggested hypoxia as a risk factor for developing glaucoma in the presence of normal intraocular pressure (IOP) [24, 25]. Taken together with the results of the present and other studies [26, 27] linking SAS and glaucomatous optic neuropathy, a positive connection emerges. A specific suggestion of the connection between normal tension glaucoma (NTG) and sleep apnea syndrome has been suggested recently [28].

Contrary evidence, however, does exist. The results of a report on records from the BVAMC of a smaller number sleep apnea patients suggested that SAS is not a risk factor for developing glaucoma [12]. These investigators used a single code for SAS and compared the risk of *emergent* glaucoma over a five-year period making direct comparison difficult. However, our data included patients whose diagnosis of sleep apnea was broader and cross-sectional. In addition, our patients had a *concomitant* diagnosis of glaucoma. 

The cross-sectional study by Geyer failed to find a relationship between SAS and the diagnosis of glaucoma [11]. In that study, 228 patients were examined specifically for respiratory disturbance index (RDI) to establish the diagnosis of SAS. The glaucoma diagnosis was based on IOP, optic disc evaluation, and visual field data [11]. A potential explanation for the discordance among these results is that early diagnosis and treatment of SAS using cPAP therapy is beneficial in reducing the risk of developing the consequences of reduced blood flow to the optic nerve head, disturbances of autoregulation, or intraocular hypoxia [29]. Others, however, have reported increased mean IOP with cPAP use [30]. This report consisted of 21 patients with SAS whose IOP was measured every 2 hours over a 24-hour period on two separate occasions and in the supine position during sleep [30].

The present study investigated nearly 71,000 records and to our knowledge represents the largest population review conducted to date regarding the association of glaucoma and SAS. 

 Prevalence for SAS (3.8%) in this study is consistent with that of other reports between 2% of the female and 4% of the male population [31]. The present study consisted of approximately 90% males and therefore may be skewed toward higher prevalence. 

The likelihood of glaucoma is known to increase with age [20]. The range is estimated to be between 0.7% of those 40–49 years of age to 3.9% of those 70–79. Other studies place the range of POAG specifically at between 1.7% and 3.0% of the Caucasian population [32–36]. The prevalence of glaucoma among patients in the present study was 5.1% (3638/70,960). This is higher than general population estimates and may be explained by including POAG as well as NTG. Alternative explanations include the fact that the population studied is generally older and perhaps at greater morbidity risk as they represent outpatients attending a clinic setting. So some selection bias may exist. 

Other limitations are inherent to this data collection. Diagnostic codes were chosen with the presumption of accuracy because they were entered by those charged with treating and managing the patients. 

Considering just the prevalence of glaucoma in individuals diagnosed with SAS, our rate is 8.4%. This is consistent with the reported by Mojon et al. (7.2%) in a smaller series which included both primary open-angle and normal-tension glaucoma [5]. 

In conclusion, the statistical results of the present study suggest a link between patients diagnosed with SAS and those diagnosed with glaucoma. The odds ratios suggest that patients diagnosed with SAS are at greater risk for having glaucoma. We recommend that questions concerning sleep-disturbed breathing be included for patients diagnosed with or suspected of having glaucoma, especially NTG or NTG suspects. Similarly, we recommend that patients diagnosed with sleep-disturbed breathing be evaluated for glaucoma. 

Further research is planned on a larger scale to confirm these results. In addition, investigation to specify pressure-dependent versus pressure-independent optic-nerve damage is planned. 

## Figures and Tables

**Table 1:** Crosstabs of SAS and glaucoma diagnosis.

	Glaucoma −	Glaucoma +	Total
Apnea −	64,825 (95.0%)	3,410 (5.0%)	68,236
Apnea +	2,497 (91.6%)	228 (8.4%)	2725

Total	67,322	3,638	70,960

Note: row percentages are in parentheses ().
